# PV to reduce evaporative losses in the channels of the São Francisco’s River water transposition project

**DOI:** 10.1038/s41598-024-56952-z

**Published:** 2024-03-21

**Authors:** Uri Stiubiener, Adriano Gomes de Freitas, Janne Heilala, Igor Fuser

**Affiliations:** 1 Graduate Academic Program on Energy, The Federal University of ABC – UFABC, 5001, dos Estados Avenue, Twr 1, Santo André, SP 09210-170 Brazil; 2 Faculty of Engineering, Monash University, Wellington Rd, Clayton, VIC 3800 Australia; 3 Faculty of Technology, The University of Turku, Turun Yliopisto, 20014 Turku, Southwestern Finland Finland

**Keywords:** Water preservation, Sustainability, C-free power generation, Solar canals, Efficiency improvement, Engineering, Energy infrastructure, Energy grids and networks, Environmental impact

## Abstract

Open water transposition channels in hot and arid regions, like those in the São Francisco River Integration Project (PISF) in Brazil, suffer significant water losses through evaporation. This paper proposes covering these channels with photovoltaic (PV) panels to reduce evaporation while simultaneously generating clean energy. The research aims to quantify water savings and energy generation potential across all channel lengths and assess whether the generated solar power can substitute grid electricity for powering the transposition pumps during peak hours, thereby enhancing energy efficiency. This study analyzed the state-of-the-art of PV generation and calculated their solar potential. Identified the specific characteristics of PISF channels and watercourses considering the regional geography, meteorology, irradiation, and social peculiarities. And, finally, assessed the feasibility of covering the watercourses with solar panels. The results reveal that covering all current PISF channels with PV panels could save up to 25,000 cubic meters of water per day, significantly contributing to water security and improving the quality of life for the local population. Additionally, the project could generate 1200 gigawatt-hours of electricity annually, meeting the energy demands of the transposition pumps during peak hours and promoting energy efficiency within the project. This research paves the way for utilizing PV technology to address water scarcity challenges and enhance the sustainability of water infrastructure projects in arid regions worldwide.

## Introduction

### Objective

This research aims to evaluate the potential water savings achieved by covering water courses with photovoltaic solar panels. Additionally, it will assess the improvement in energy efficiency of the São Francisco River Integration Project (PISF) by utilizing solar energy to power its pumping system during daylight hours.

### Background

The PISF addresses a historical demand for water resources in Northeastern Brazil. This region houses 28% of the population with access to only 3% of the country’s total water resources. The São Francisco River supplies 70% of the region’s water, historically impacted by severe droughts. Projected climate change and global warming scenarios suggest prolonged and intensified drought periods, highlighting the critical importance of water security for the region.

### The problem

**Figure 1:**
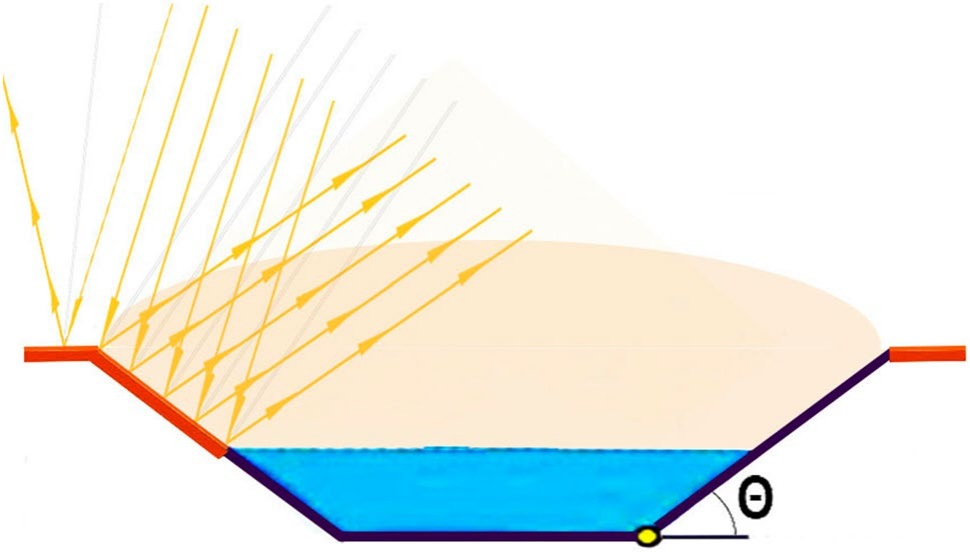
Hot zone due to the sun radiation on channel’s concrete borders.

PISF transfers water from the São Francisco River to surrounding arid areas. However, solar radiation directly heats the water and adjacent concrete walls, of the trapezoidal shape channels leading to increased evaporation through a “hot zone” effect over the channel (Fig. 1) due to mirror effect.

Water loss is calculated based on the open water surface area and local evaporation rates. Circa 30% of the captured water is lost through evaporation. PISF’s components are subjected to the same climate conditions and environmental variables. The volume of water lost by evaporation *EVP* was obtained from Eq. (1) where $A_{(i)}$ is the free water surface and the local evaporation rate obtained from the National Institute of Meteorology (INMET) measuring stations is $evp = 3,150~{mm/year}$.

1 $$\begin{aligned} EVP = {\sum {{EVP}_{ (i)}}} = \sum ({A_{ (i)}}\times {evp_{ (i)}}) = {evp}\times \sum {A_{ (i)}} \end{aligned}$$

At the North Axis, 16.4 $m^3/s$ of water is captured from the river. Approximately 5.4 $m^3/s$ is lost through evaporation (90% of these in the reservoirs), leaving only about 11 $m^3/s$ with a useful destination (distribution to supply the population)^1^. At the East Axis, 10 $m^3/s$ of water is captured and only about 7 $m^3/s$ supplies the population.

De Farias, Curi, and Diniz calculated water losses using the AcquaNet simulation model in several scenarios and analyzed the performance of PISF’s East Axis serving the Paraíba River Basin. They found total loss ranging from 48 to 60%. Of this, 3.5% to 6.9% were attributed to evaporation. Leakage and in-transit losses respond to complete the total loss^2^.

### As water is the subject of this project, initiatives to reduce losses due to evaporation must be considered

### Proposed solution

By covering water courses with photovoltaic solar panels, this research seeks to achieve two key objectives:

- Reduce water evaporation: The panels will provide shade, significantly reducing water loss through evaporation.
- Generate clean energy: The installed solar panels will generate electricity, potentially powering the PISF pumping system during the day, reducing reliance on grid-based power, and contributing to energy efficiency.

### Significance

This research can contribute to addressing the water scarcity challenges in Northeastern Brazil by demonstrating the potential of water-saving technologies and promoting sustainable energy solutions for water infrastructure systems. The findings can inform policy decisions and guide the implementation of similar projects in other regions facing water scarcity and renewable energy integration opportunities.

##  Method

High water loss from critical infrastructure highlights the importance of addressing evaporation through innovative solutions like covers to improve sustainability. Further research on mitigation strategies is crucial for responsible water resource management. To achieve effective mitigation strategies, the research follows a three-step process:

- Literature review: A comprehensive review of academic and industry literature is conducted to establish the current and future outlook for photovoltaic solar energy generation in Brazil and globally.
- Characterization of PISF: The structure and characteristics of the channels and watercourses within the PISF are identified and documented.
- Geographic and meteorological characterization: The geographical and meteorological conditions of the region are analyzed to better understand the potential impact on the proposed solution.

### Evaluation of PV arrays coverage

The research then explores the potential of using photovoltaic arrays to cover watercourses and assess its feasibility for PISF. This involves three key steps:

1. Coverage area and energy generation: The coverage areas for the photovoltaic arrays $({S_ {PV}})$ are determined. The surface to be covered by PV arrays $({S_{PV}})$, is obtained from Eq. (2) where $P_{PV}$ is the peak power of the PV installation, $\mu _{PV}$ is the power density. This surface was called “Solar Area”.
  2 $$\begin{aligned} S_{PV} = {\frac{P_{PV}}{\mu _{PV}}} \end{aligned}$$
2. Energy generation: The expected annual energy generation for PV installations $({E_{PV}})$ is obtained from Eq. (3), where ${\overline{H}}$ is the annual average of, locally measured, daily solar radiation (in $kWh/m^{2}.day$), $\eta _{PV}$ is the rated efficiency of the PV panels and *PR* is the performance ratio of the PV power system.
  3 $$\begin{aligned} E_{PV} = 365 \times \overline{H} \times \eta _{PV} \times PR \times {S_{PV}} \end{aligned}$$
  In this paper, was assumed that $\mu _{PV} = 1\,MW/10,000\,m^2$, $\eta _{PV} = 15\%$, and $PR = 0.8$.
3. Water savings: The annual water savings $({Q_ {PV}})$ associated with the coverage area $({S_ {PV}})$ are estimated based on relevant references^3,4^.

Through this comprehensive approach, the research seeks to evaluate the effectiveness and feasibility of employing photovoltaic arrays to reduce water loss and enhance sustainability within the PISF.

## Literature review

### Water scarcity

According to the United Nations Development Programme (UNDP), the Human Climate Horizons (HCH) report states: *“[...] the weather conditions we experience are changing, resulting in higher year-round temperatures, more extremely hot days resulting in higher year-round temperatures, more extremely hot days [...]”*^5^.

This predicts that severe drought and lack of water resources will persist in the region. Warming influences human development and all spheres of our lives, including our health, livelihoods, and ability to work. Water availability is of crucial importance for the local population.

### Impacts related to PV installations on water

PV installation on water requires further description and analysis, when the artificial channels were designed and built, the environmental risks were considered^6^. If only channels are covered the impacts on aquatic flora and animal life will be less. However, to shoreline installations, other PISF-free water surfaces, natural rivers, and reservoirs will be covered by floating PV installations, as shown in Fig. 8. In that case, a new study of environmental impacts and risks should be carried out and approved by the respective legal entities and authorities, extending their installation, changing climate, and synergizing with wave power potential and stakeholders.

Alongside the technical and economic aspects, the socio-environmental aspects must be considered when designing PV installations^81^. Some of these aspects are related to visual impacts, facility safety, impacts on tourism and leisure, impacts on water quality, impacts on aquatic flora and animal life, impacts on bird habitats, etc. Some of these impacts are^7^:

#### Visual impacts

The large areas occupied by PV installations will bring aesthetic and visual impact. They are subjective and personal. Some people like it, others don’t. Studies indicate that reservoirs covering up to 50% of the wetland area by PV arrays are acceptable in other parts of the world^8^.

#### Safety

High voltage DC power cables, with tension levels of 1.5 *kV*, used in utility-scale PV plants are relatively near the floor level. The security issue has two aspects: the safety of people and animals in the vicinity of the plant and the safety of installations regarding the physical impacts or short-circuiting effects due to foreign bodies. Currently, there are no technical standards that address these issues. However, it is necessary to establish a region of restricted circulation around the PV array. Equal importance should be given to the training of a skilled technical workforce to work with PV facilities.

#### Jobs, tourism, and leisure

These impacts result from the two previous items: the social perception of the beauty of the installation and the security perimeter. Transposition channels are not leisure areas. Rivers, lakes, and reservoirs should be studied in accordance with the multiple uses of these water bodies and the appropriate regulations.

Clean water flow in PISF channels can host fishing farms, introducing farmed aquaculture as a new activity in the region reducing poverty and increasing the local employment and food security^3^. PV will bring new job vacancies to the neighborhood. The operation and maintenance of PV facilities require technically educated people for the necessary operations. Other job openings, as well as indirect jobs and services needed to serve new employees, and their families, will emerge.

#### Water quality

Some water quality parameters may be more affected by covering channels, whereas others are slightly affected. Water pH, total dissolved solids, electric conductivity, and alkalinity are less affected. Dissolved oxygen (DO) and algae concentration, seem to be significantly affected by channel coverage. As a result, the nutrients and phosphorus are impacted, as they are related to algae concentration in water.

El Baradei and Al Sadeq investigated the water quality in channels covered by PV arrays. They found that the channel coverage will not harm algae, nutrients, phosphorus, pH, and alkalinity. They found that the ideal rate of covering the Sheikh Zayed Canal, in Egypt, to optimize the power production, water quality, and water loss through evaporation, is between 33 and 50%^9^. They suggest that a water quality simulation should be done, for every “solar canal” of fresh or irrigation water project, in order to meet the water quality standards.

Floating PV arrays are relatively recent facilities, all less than 10 years old. No negative impact on water quality has been reported in these facilities to date^10^. According to Rosa-Clot and Tina^8^, materials in contact with water are not polluting. Reports demonstrate that shading does not affect, or even benefit, the water quality^10,11^.

####  Vegetation

The transposition channels have no vegetation and the water must not be contaminated with organic matter. PV arrays on natural water surfaces shade but do not block the incidence of light on the water surface. Shading of the aquatic surface may have effects on vegetation and micro-algae in the reservoirs^3,11,12^. It has been found that PV array alone, or with the installation of aerators and/or underwater ultrasound systems, reduces the proliferation of algal blooms in waters with many nutrients, thus preventing staining and the bad taste of the water^13^.

####  Animal life

Fishing farms can be introduced in the PISF channels as a new aquaculture activity^3^. In ponds containing fish, no harmful effects on aquatic fauna have been reported due to shading caused by PV arrays. On the contrary, in at least one case, in the lake with fish (mainly carp), it was found that fish prefer shaded water with little ripple under the PV array. There was a great increase of the shoal in the region of the PV arrays making the surroundings a very popular place for fishermen^8^.

It has been observed that the coverage of the already existing flooded areas did not alter the migratory habits and migratory routes of the birds in the region. However, as floating PV installations are recent, it is necessary to observe the local biome of each installation to identify and mitigate any environmental impacts. There are no conclusive studies on the environmental impacts caused by photovoltaic installations. The research into environmental impacts must be continuous, thorough, and comprehensive. Likewise, the socioeconomic impacts of such projects must be evaluated.

###  PISF transposition system description

The whole system includes, besides the artificial open-top channels and pumping stations, the natural waterways of the rivers and reservoirs. Artificial channels comprise 5% of the free water surface, riverbeds 5%, and reservoirs 90%^1^.

At the current stage, all artificial system sections are operational and reach the most distant users. Retrospectively looking, civil works were completed in 2017 (East) and 2020 (North)^14–16^. Built power infrastructure delivers to 9 pumping stations and substations but must still be fully equipped. Only the two installed pumping systems attend the current flow rates in each station; without a standby pump.

####  The channels

**Figure 2:**
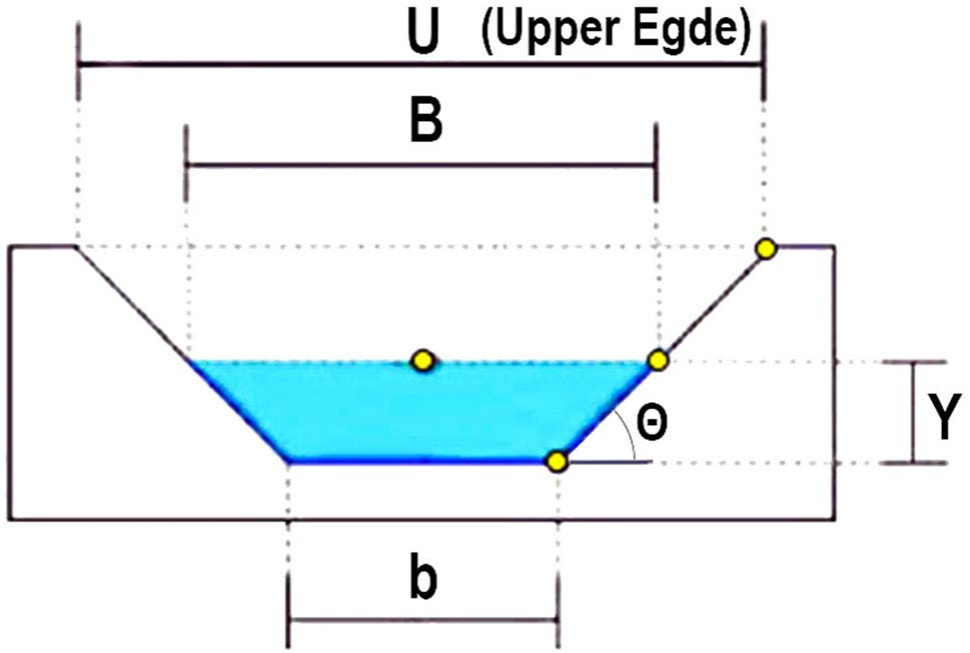
Typical channel cross-section. The size parameters *U* is the upper edge dimension and *b* is the bottom edge of the channel. The water blade with a deep of *Y* results in the water surface with an extension $\underline{B}$.

The channels are artificial constructions of trapezoidal shape with typical cross-sections as illustrated in Fig. 2. The parallel edges of the channel allow it to be used as a support base for metal structures perpendicular to the channel, which can accommodate the PV modules.

At the project location’s latitude, the ideal slope of the panels is 9 degrees (almost horizontal), which requires a short distance to avoid shading one line of the panel over the next, reducing the space between the lines and allowing for more compactness. The inclination of the modules must be such that it allows for the flow of water used in the cleaning of the modules. Typically 10–15 degrees allow for adequate drainage. At this latitude, there are no great advantages in using vertical solar tracking systems, which reduces installation, as well as maintenance, costs.

On the Northern Axis of the project, the channels are naturally oriented to the north, so that the channel cross section accommodates the PV arrays in the ideal orientation. On the Eastern Axis, the modules can also be mounted on structures transverse to the channel direction, in-spite they will not be oriented to the north.

**Figure 3:**
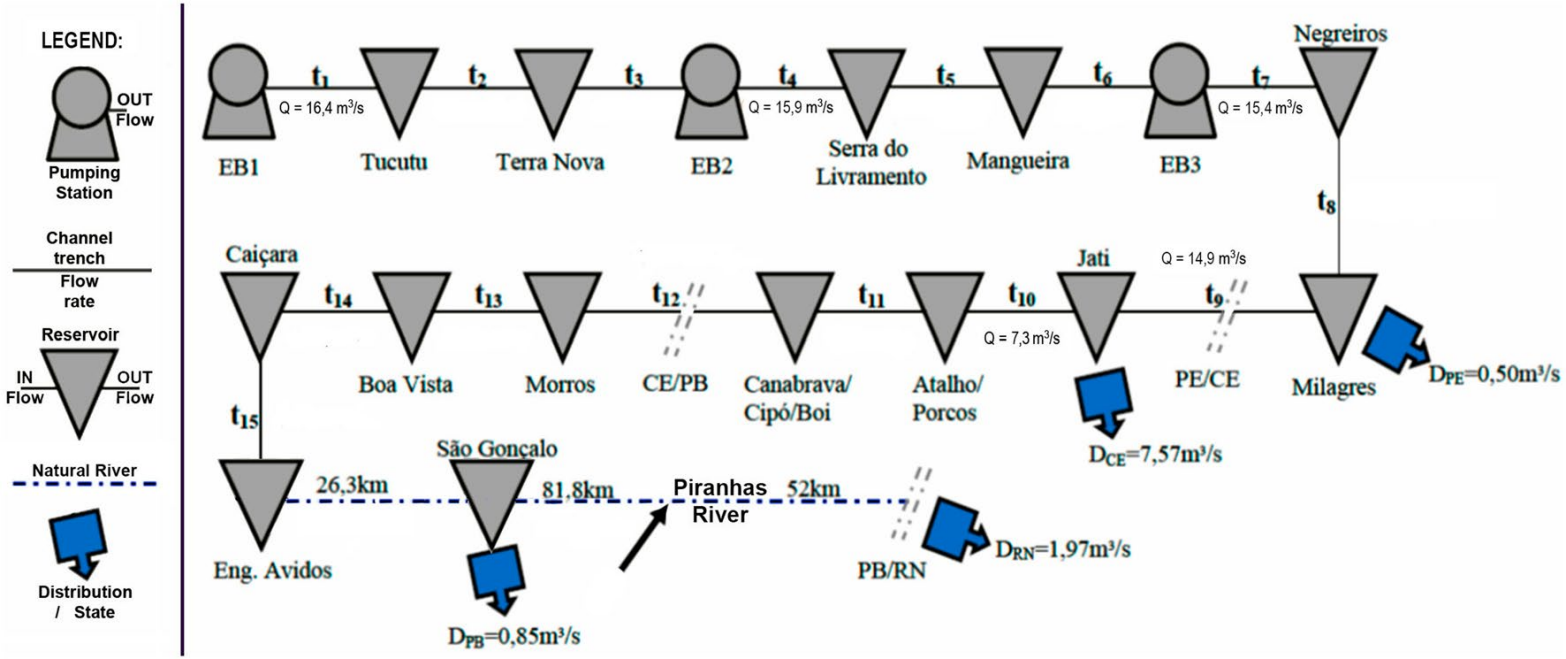
The Northern Axis schematic diagram.        Source: Trajano Jr. ^1^.

The flowchart Fig. 3 shows the structure of the Northern Axis of the PISF: the pumping stations, the current flow rates in each stretch, the position of the reservoirs, and the water consumption points ($D_{UF}$) with their respective outflows. However, these outflows were leveled to attend only 85% of current water demand at the delivery points^17^.

The project schedule was divided into 3 stages called “Goals”. The channels of the first stage of the North Axis (Goal 1 North), up to the Jati Reservoir, have 20 *m* upper-edge and an extension of 110 *km* as described in Table 1.

**Table 1:** Channels of Northern Axis.

	From	To	U-Edge (*m*)	Lenght (*m*)	Area ($m^{2}$)
TN00	São Francisco River	EBI-1 water intake	20	2.060	41.200
TN01	EBI-1 outflow	Tucutú Reservoir	20	6.578	131.560
TN02	Tucutú outflow	Terra Nova Reservoir	20	34.480	689.600
TN03	Terra Nova outflow	EBI-2 water intake	20	2.249	44.980
TN04	EBI-2 outflow	Serra do Livramento Reservoir	20	1.433	28.660
TN05	Serra do Livramento outflow	Mangueira Reservoir	20	17.340	346.800
TN06	Mangueira outflow	EBI-3 water intake	20	3.470	69.400
TN07	EBI-3 outflow	Negreiros Reservoir	50	388	19.400
TN08	Negreiros outflow	Milagres Reservoir	20	20.415	408.300
TN09	Milagres outflow	Jati Reservoir	20	21.940	438.800
Goal 1 N		Sub-total		110.353	2.218.700
+					
Goal 2 N	Jati Reservoir	Caiçara Reservoir	12	108.100	1.297.200
+					
Goal 3 N	Caiçara Reservoir	Piranhas river	10	52.000	520.000
		Northen axis total		270.453	4.035.900

Source: Data from Brazil–Interior Ministry, 2006

Goal 2 and Goal 3 channels are smaller but longer, totaling 160 *km* up to São Gonçalo Reservoir at Piranhas River.

Table 2 describes the Eastern Axis, which is also scheduled in three goals, distinct in shape and construction schedule. The channels of Goal 1 East, up to the Copiti Reservoir, have 15 *m* upper edge and an extension of 93.4 *km* including pumping stations EBV-01 to 04, and they were built first. Goal 2 East and Goal 3 East channels have 10 *m* upper-edge and an extension of 88 *km*. These channels, the two pumping stations, EBV-05 and 06, and the Monteiro tunnel were built later. Eastern Axis is complete and operational.

**Table 2:** Channels of Eastern Axis.

	From	To	U-Edge (*m*)	Lenght (*m*)	Area ($m^{2}$)
TE00	Itaparica reservoir	EBV-01 water intake	15	68	1.023
TE01	EBV-01 outflow	Areias Reservoir	15	6.746	101.197
TE02	Areias Reservoir	EBV-02 water intake	15	1.372	20.579
TE03	EBV-02 outflow	Braunas Reservoir	15	2.038	30.572
TE04	Braunas Reservoir	Mandantes Reservoir	15	11.250	168.749
TE05	Mandantes Reservoir	EBV-03 water intake	15	1.214	18.205
TE06	EBV-03 outflow	Salgueiro Reservoir	15	1.374	20.611
TE07	Salgueiro Reservoir	Muquém Reservoir	15	30.322	454.830
TE08A	Muquém Reservoir	Jacaré aqueduct	15	9.933	148.999
TE08B	Jacaré aqueduct	Cacimba Nova Reservoir	15	10.325	154.871
TE09	Cacimba Nova Reservoir	EBV-04 water intake	15	865	12.9751
TE10	EBV-04 outflow	Bagres Reservoir	15	5.243	78.650
TE11A	Bagres Reservoir	Caetitu aqueduct	15	10.485	157.275
TE11B	Caetitu aqueduct	Copiti Reservoir	15	2.204	33.058
Goal 1 E		Sub-total		93.440	1.401.595
+					
TE12	Copiti Reservoir	Moxotó Reservoir	10	40.300	403.000
TE13	Moxotó Reservoir	EBV-05 water intake	10	6.100	61.000
TE14	EBV-05 outflow	Barreiro Reservoir	10	4.500	45.000
TE15	Barreiro Reservoir	EBV-06 water intake	10	1.600	16.000
TE16	EBV-06 outflow	Campos Reservoir	10	6.800	68.000
TE17	Campos Reservoir	Barro Branco Reservoir	10	5.900	59.000
TE18	Barro Branco Reservoir	Mangueira Reservoir	10	10.400	104.000
Goal 2 E		Sub-total		75.600	756.000
+					
Goal 3 E	Mangueira Reservoir	Poções Dam	10	12.000	120.000
		Eastern axis total		181.040	2.277.595

Source: Data from Brazil–Interior Ministry, 2006

The top edge area of all channel extensions totals 6.313.495 $m^{2}$. The channel’s upper-edge design can easily support steel structures for PV coverage. All the 450 *km* of the open channels extension are ready to receive PV arrays.

####  Pumping stations

**Figure 4:**
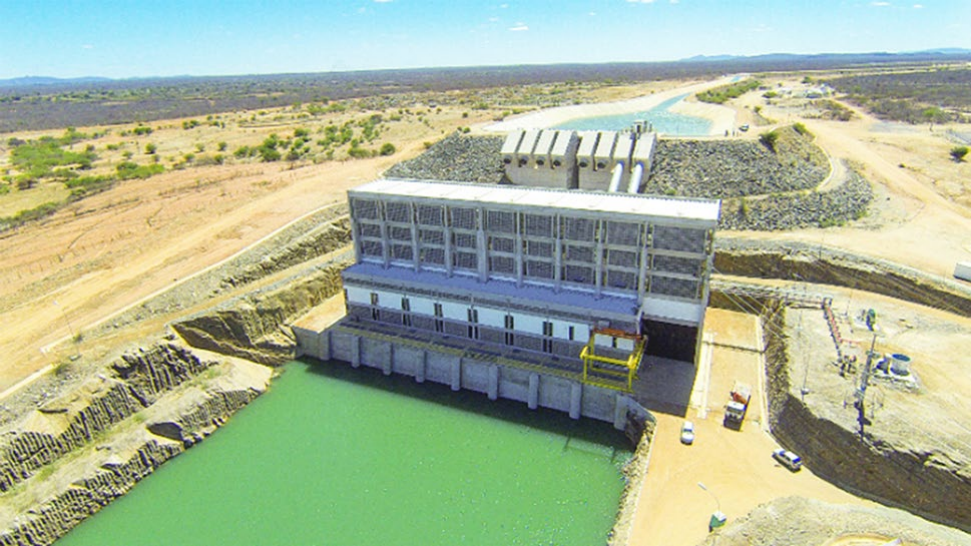
Pumping station EB-1.    Source: Brazil---Ministry of National Integration/Disclosure.

The three pumping stations EB-1, EB-2, and EB-3 on the North Axis are designed for maximum future capacity and can accommodate up to 8 pumps each. In the current phase, to meet the project’s flow rate, only 2 pumps were installed at each station. The pumps used in EB-1 (Fig. 4) are KSB model SEZ 15-110/2 driven by 5,500 *kW*/ 6,900 *V* electric motors^18^. At EB-2 the pumps have 10 *MW* motors; on EB-3 motors are 2.7 *MW*. Altogether, the 3 pumping stations on the North Axis require 36.5 *MW* in the current stage, and 146 *MW* in the final phase of the project.

On the East Axis, the flow rates are lower. About 60% of flow rates in the North Axis as foreseen in ANA–Resolution No 632018. Water must be pumped to cover greater unevenness, reaching a total of 332 m. This will be done in six steps, so 6 pumping stations, EBV-1 to 6, are built. Each of these will have 4 pumps to reach the final stage flow rate; in the first stage – only 2 pumps at each station.

The power of the designed pumps is 5.3 *MW*, 3.7 *MW*, 5.5 *MW*, 5.3 *MW*, 2.2 *MW*, and 3.4 *MW*, respectively. Altogether, pumping stations on the East Axis require 51 *MW* in the current stage, and 102 *MW* in the final stage of the project.

To supply power to all the pumps, new 230 *kV* transmission lines were designed and built to serve the final project’s stage. The 270 *km* of high-voltage power lines are connected to the Paulo Afonso Hydroelectric Power Plants Complex which contains a total of 23 turbo generators with a nominal installed capacity of 4.28 *GW*^19^. Nine substations reduce the tension to 6.9 *kV*, as required by the pumps’ electric motors.

### Regional meteorological and social characteristics

The Northeast hinterland is the driest region of Brazil^20^ with precipitation not exceeding 400mm per year in different locations and, it is, therefore, susceptible to desertification^21^. Semiarid regions are subject to water shortages and soil degradation in such places is likely to increase with climate change^22^. The watercourses are generally formed by temporary rivers (also called intermittent), except for the São Francisco River.

According to the United Nations Convention to Combat Desertification^23^, the determination of an area susceptible to desertification can be carried out through the aridity index (AI).

This index corresponds to the ratio between precipitation (*mm*) and potential evapotranspiration (*mm*), the latter being defined as the amount of water that could evaporate or transpire from a vegetated surface due to atmospheric influence^24,25^.

**Figure 5:**
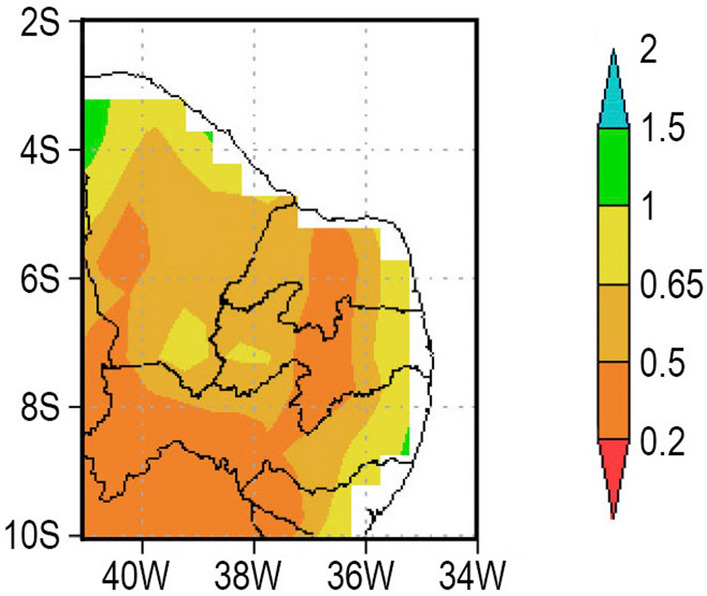
Northeast Brazil aridity map, showing the PISF region in which AI is 1.0 and below. Source: Adapted by the authors from Reboita et al.^20^.

When the AI is between 0.2 and 0.5 the climate is characterized as Semiarid (BSh in Köppen climate classification)^26^; what is the situation in large areas of the Northeast hinterland as shown in Fig. 5 which covers all of PISF’s region. The semiarid region of Northeast Brazil covers 969,589 $km^2$^27^.

The Brazilian Semiarid is the most populated semiarid area in the world and, due to climate adversities, associated with other historical, geographical, and political factors, that date back hundreds of years; it houses the poorest part of the Brazilian population, with the occurrence of serious social problems^27,28^.

This region has one of the highest rates of social and economic inequality in Brazil. Problems such as hunger, poor distribution of income, poverty, and rural exodus are recurrent, above all, in the interior cities of this deprived region. This has caused large-scale migrations.

In this area, which covers the eastern portion of Piauí (PI) and most of the states of Ceará (CE), Bahia (BA), Sergipe (SE), Pernambuco (PE), Paraíba (PB), Alagoas (AL) and Rio Grande do Norte (RN), the main economic practice is extensive livestock.

Therefore, in this region, known as the “Polygon of Drought”, the problem of lack of rain has been known and documented since the 16th century. It was officially first created in 1936. By the 1946 Constitution, Article198, the execution of a defense plan against the effects of the so-called drought in the Northeast was regulated and disciplined. The legal framework for the creation of this area was finally instituted in 1968, through the Brazilian government Act No 63,778^29^.

Contrary to popular belief, the northeastern drought is not solely a natural phenomenon. While seasonal shifts in rainfall patterns and occasional delays in precipitation play a role, several factors contribute to its severity and persistence. This usually happens when the Inter-tropical Convergence Zone (ITCZ) does not reach the northeastern region in the period between summer and autumn. Factors such as *La Niña* and the burning of native vegetation also directly affect the region’s drought regime.

According to the WMO, *La Niña* will continue to affect temperature and precipitation patterns and exacerbate droughts and floods in different parts of the world. Terrestrial water storage (TWS) of the São Francisco river basin exhibits a gradual decrease, with 2021 being the year with the lowest TWS between 2002 and 2021^30^. For the first time this century, the La Niña phenomenon will last for three consecutive years.

The year 2021 was ranked between the fifth and seventh warmest year on record, with the global annual mean temperature of 1.11 $\pm 0.13 ^\circ$C above the 1850–1900 pre-industrial average, despite prevailing La Niña conditions^31^. The last ten years were the warmest on record, with rising sea levels and warming oceans accelerating. The continuity of *La Niña* prolongs the conditions of drought and flooding in the affected regions it caused the longest and most severe drought in recent history.

Drought periods directly affect the weather of this area. Exerting pressure on water, agriculture, and food supply, climate change is having devastating consequences for arid regions^32^. The global warming scenario points to prolonged and severe drought periods^33–35^, which will lead to prolonged and greater suffering for the population of this arid region. Drought periods make water supply to this region of vital importance.

###  PV power potential

**Figure 6:**
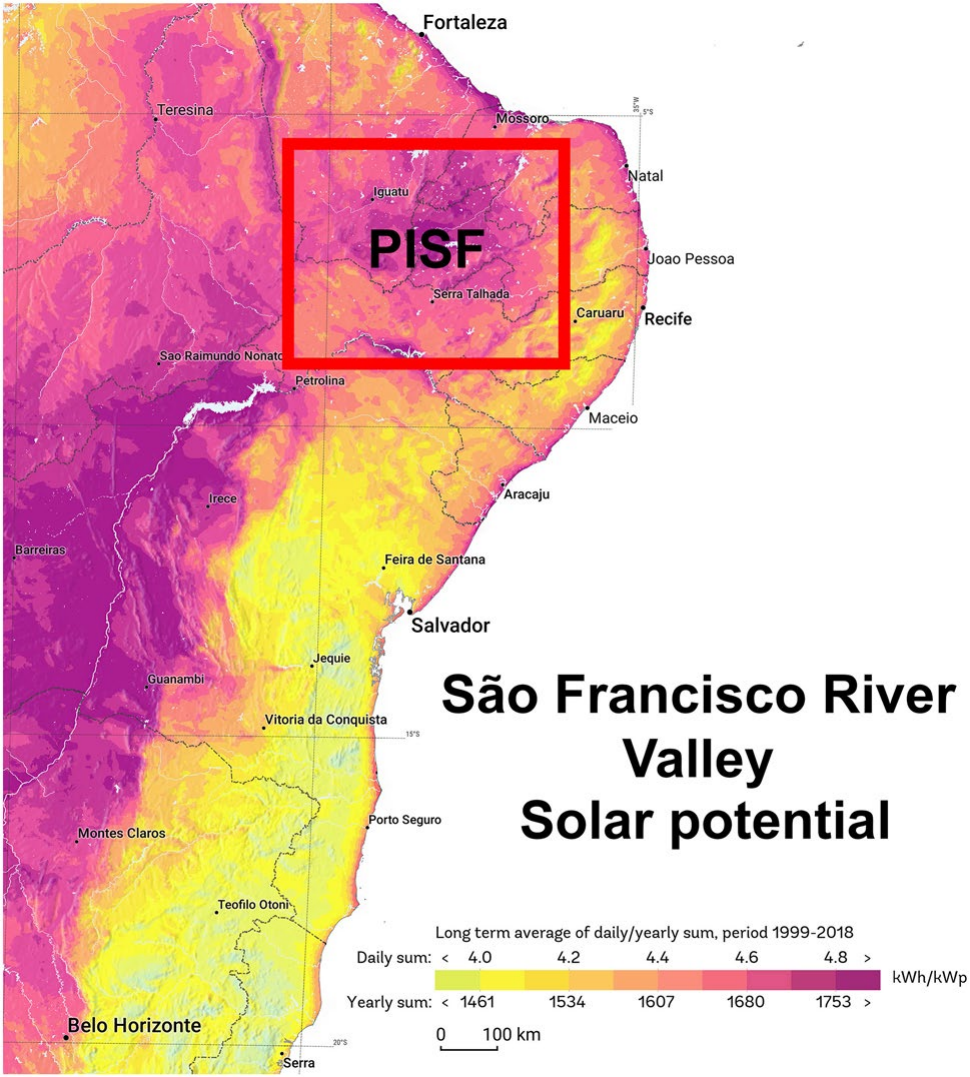
Solar potential of all the São Francisco Valley extension, highlighting the region of the PISF water transposition project. Source: Adapted by the authors from globalsolaratlas.info.

The Northeast region has the largest solar energy resource, on average 5,9 $kWh/m^{2}$, and presents the smallest inter-annual variability (between 5.7 and 6.1 $kWh/m^{2}$)^36^. The high solar radiation encourages using PV as a power source. Fig. 6 illustrates the solar potential of the São Francisco River valley and highlights the PISF area.

Traditional PV arrays installed on land require roughly 3.3 *ha*/*MWp*. This is because spacing is necessary to prevent shading of one row of panels by the next, and to allow for access for cleaning and maintenance. In contrast, floating PV installations are denser and can occupy less area than their terrestrial counterparts. Floating PV footprint is smaller than on land PV arrays In this article the $\mu _{PV} = 10\,{m^2/kWp} = 1\,{MWp/ha}$ was considered for both^37,38^.

Channels top can be fully occupied by PV modules separated only by walkways between the lines to allow maintenance, and some required access to the channel itself. Those why the considered $\mu _{PV}$ value is conservative^39–42^ and is used to safely estimate channels and free water coverage area by PV panels. Current solar technologies allows $\mu _{PV} = 7.5\,{m^2/kWp}$. When detailing the channel coverage project, engineering design can reach this lower occupation factor and increase the power installed.

## Results and discussion

###  Solar radiation

**Figure 7:**
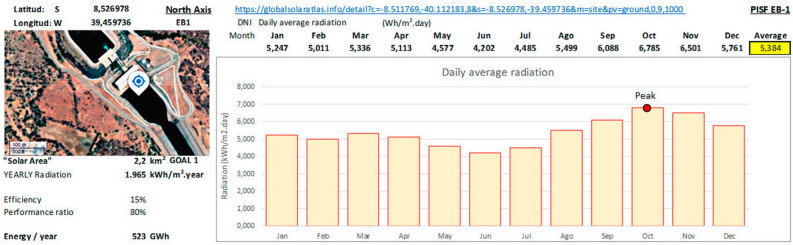
Local radiation profile at Pumping station EB-1. Source: Elaborated by the authors using the data of the Global Solar Atlas^43^.

The seasonal profile for PISF’s location is in Fig. 7. According to monthly data from the Global Solar Atlas, at EB-1 (8.526978 S; 39.459736 W) the annual average of daily radiation is 5.384 $kWh/m^{2}.day$. The peak occurs in October and reaches 6.785 $kWh/m^{2}.day$.

###  Feasibility of using PV energy

To meet the power requirements of the 2 pumps installed in each pumping station of the Northern Axis, 365,000 $m^2$ of solar panels are required. Considering the typical upper edge width of 20 *m* of the channels close to the pumping stations, it is necessary to cover an 18 *km* channel extension in the vicinity of the three pumping stations (can be upstream or downstream). This will reduce transmission losses and minimize the cost of cabling. It is necessary to cover less than 17% of the Goal-1-North 110 *km* length of the built channels. To meet the final (maximum) capacity of the project, this percentage rises to 68%. However, Goals 2 and 3 of the northern axis have an additional 160 *km* of channels with an upper edge width of 12 and 10 *m*.

At the Eastern Axis, with channels extension of 201 *km*, to meet the power requirements of the 2 pumps already installed in each of the 6 operating pumping stations, 508,000 $m^2$ of solar panels are required. As the typical upper edge width of 15 *m* of the channels, it is necessary to cover 34 *km* of channels which is circa 21%. This percentage rises to 42% to meet the final stage of the project.

The PV array will cover the entire channel, shading the water regardless of the flow and depth of the water. For this purpose, the Indian model, with the solar panels mounted on a metallic structure supported on the upper edges of the channel, may be the most suitable. The PV installations can reduce by 25% the daily consumption of the grid energy to power the pumps.

This design concept was successfully used to cover 750 *m* of an irrigation channel in Narmada, in the state of Gujarat, India, 2013, reducing water loss through evaporation and generating 1 *MW*^44^, p.19. The same concept of installation, including the substation, can be observed in an article regarding megawatt-scale canal-top solar power plants bid in India, with a photo of the area view^45^.

California, USA, grapples with comparable issues concerning its water channels. The region experiences severe periodic droughts, exacerbated by climate change and excessive water consumption. These factors wreak havoc on the region’s water supply. Open irrigation channels further exacerbate the problem by allowing precious water to evaporate. Despite this, the intricate network of channels remains crucial for delivering water throughout California. The channels snake their way through the Central Valley, transporting water to irrigate crops. The sheer scale of this system, spanning 6,500 *km*, presents an opportunity for covering the aqueducts with PV systems. This emerging technology addresses the interconnected water-energy-food nexus, offering a promising response to the challenges faced by California’s water infrastructure. Researchers from UC Santa Cruz and UC Merced found that *“...it makes sense to cover channels with PV panels because renewable energy and water conservation are a win-win project...*^46^ *...Energy and water co-benefits from covering channels with solar panels”*^46,47^.

Balram and Narayan Bhardwaj suggested the use of micro-hydrokinetic turbines combined with PV panels chancels’ to increase power generation. PV panels on the topside capture the sun’s energy, while micro-hydrokinetic turbines on the bottom capture hydro-power from the flowing water^48^. This combination increases the power output of both the solar and hydro components. The channel-spanning infrastructure to support the PV arrays also serves as a solid foundation for the hydrokinetic turbine arrays^48,49^.

Taboada et al. research in Northern Chile, at Antofagasta in the Atacama Desert, found that the water evaporation reduction in a pond with floating covers in their region was at least 90%, compared to an uncovered pond^50^. Recent studies pointed to an average reduction of the evaporation from open water bodies in studied semi-arid regions up to 60%^10^. The authors consider that these rates may be too high for the PISF project. A field investigation on PISF channels should be done to accurate the regional value of the evaporation reduction rate.

Abd-El-Hamid et al. have been researching the evaporation loss of Lake Nasser, in Egypt. Their study suggests the use of floating PV arrays to cover parts of the lake to reduce water losses due to evaporation. They found that covering the very shallow parts of the water body, up to 1.0 *m* depth, will provide the highest water saving^51^.

Pringle, Handler and Pearce suggested that when covering the water surface with PV arrays, a reduction of 20% to 25% in the loss of water through evaporation is expected^3^.

PISF’s artificial channels are ideal for this solution. The surface that can be covered by PV arrays on the totality of the PISF channels is about 6.5 ${km^{2}}$ (sum of tables 1 and 2). If considering the uncovered spaces due to engineering needs, a surface area $(S_{PV})$ of at least 5 ${km^{2}}$ is available for PV installations. If the entire length of the channels is covered, the installed PV power will be 500 *MWp*, providing solar energy to the pumping stations and grid during times of significant radiation (day). The energy generated by these facilities is obtained from Eq. (3): $E_{PV} = 365 \times \overline{H} \times \eta _{PV} \times PR \times {S_{PV}} = 365 \times {5.384} \times {0.15} \times {0.8} \times {5} = 1,180\,{GWh}$. Although the combination of PV and hydrokinetic turbines technology was proposed for canal-top projects, the design can be modified and adapted to the floating solar concept environment^48^ increasing the energy produced.

Water savings can range from 15,000 to 20,000 $m^3/year$ for each installed 1 *MWp*^4^. PV power plants of 500 *MWp* will save: ${500\times [15,000~to~20,000]}/{365}$ = [20,548–27,397] ${m^3_{Water}/day}$. As the PISF region is very hot, the tendency is to stay at the top of the referenced range of values.

Despite representing only 5% of the overall water surface, artificial channels experience higher water loss compared to natural waterways, contributing to a staggering 16% of the total loss. Covering these channels is crucial to reducing water loss through evaporation.

According to the National Water Agency (ANA), water losses in the northern axis channels amount to 800 *L*/*s*^17^, while the eastern axis channels lose 500 *L*/*s*^52^. This totals 1,300 *L*/*s*. Covering the channels with solar panels offers the potential to avoid up to 25% of this loss. This significant reduction translates to a daily saving of 1,000 water truck trips, each carrying 25 ${m^3}$ of water. The implementation of this solution could significantly improve water conservation efforts, reduce reliance on water trucks, and contribute to a more sustainable water management system.

While the artificial channels of PISF account for only 5% of its total water surface area, the natural riverbeds make up a similar proportion, together covering 10% of the system. The remaining 90% of the water surface area belongs to reservoirs, ponds, dams, lakes, and lagoons (^1^. For these large water bodies, a different approach to reducing water evaporation is more suitable: floating photovoltaic islands.

There is no incompatibility between the 2 models of PV plants described, which can be combined according to engineering design conveniences.

### Floating PV installations

This method involves installing solar panels on floats and creating platforms partially covering the water surface. This approach generates clean energy while simultaneously reducing evaporation losses through evaporation in the regions covered by the solar panels^53^, contributing to increased water availability for distribution to consumption points^54^. By utilizing the same water surface for both energy generation and water conservation, this approach optimizes the use of available resources in terms of a multi-functional use of water bodies.

Studies have shown that floating systems, compared with suspended systems, have a higher yield in terms of evaporation reduction^55,56^. Floating PV also takes advantage of the water’s cooling effect and can use horizontal solar tracking to improve its efficiency^40^. Researchers pointed out that the average power production of floating PV systems is up to 15% higher than traditional on-land installations due to the lower working temperature^57^.

Floating PV systems already exist in Brazil, installed in water reservoirs and even close to hydroelectric power plants dams. At the Figueiredo das Lages farm, in Cristalina-GO, a 300 *kWp* floating PV array was installed in 2016 to reduce water evaporation and generate electricity, view photocopy^58^. This concept is used in several similar installations all over the world and is expanding fast^59^. In 2018, the floating PV technology surpassed the 1,000 *MWp* mark^60^. In 2022 the total installed capacity exceeded 4 *GWp*^61^.

**Figure 8:**
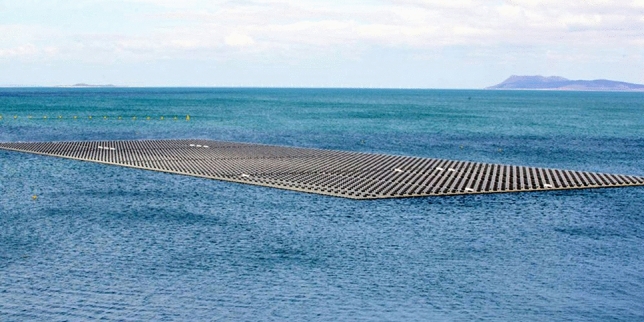
Floating PV Power Plant at Sobradinho hydroelectric power plant reservoir.to Springer Nature exclusively under CC BY SA for this study^63^.

The Sobradinho hydroelectric power plant is located in the same region as the PISF. The meteorological conditions are the same for both of them. The PV generation potential and the annual energy yield of this region are the best in Brazil’s territory. Power generation facilities up to 5.0 *MW* are regulated by a specific distributed generation (DG) legislation in Brazil. Larger facilities require new regulations. The first stage of Sobradinho’s 5.0 *MWp* floating PV power plant R &D project, a 1.0 *MWp* floating array (Fig. 8) started operation in 2019. Data obtained from this plant can lead to accurate and precise design parameters for the suggested PISF PV project^62^.

## Capital expenditure estimation

The Capital Expenditure (CAPEX) of a PV power plant in Brazil ranges between 2,500 and 5,000 *BRL*/*kWp*, with a reference CAPEX of 3,800 *BRL*/*kWp* (base 2021)^64^. These values were validated by Enel Green Power, which built the ground-mounted PV plant Bom Jesus da Lapa, in the region close to PISF, with a rated power of 80 *MWp*, running since 2017, with a declared CAPEX of 3,750 *BRL*/*kWp*.

The CAPEX of PV-FPP is slightly higher, owing chiefly to the need for floats, moorings, and more resilient electrical components. The cost of floats is expected to drop over time, however, owing to better economies of scale^60^. The CAPEX estimation of a PV-FPP in Brazil ranges between 3,800 and 6,500 *BRL*/*kWp*, with an average value of 5,000 *BRL*/*kWp*^64^.

A study based on January 2019 prices pointed to a CAPEX of 4,415 *BRL*/*kWp* and a payback of 10 years. However, when considering the investment in a floating PV system to avoid water supply to the population of the arid regions of the Northeastern hinterland by diesel-driven trucks, the payback is only 4 years^54^.

Channels coverage requires additional steel structure than a land installation but does not require land acquisition. The estimated CAPEX will be in the range between ground-mounted PV and PV-FPP, circa 4,500 *BRL*/*kWp*.

PV systems can be implemented in stages and come into operation as soon as each stage is completed, easily adapted to the budget and schedule. The final CAPEX of PISF was updated to 18 billion BRL^65^. The cost of the electric power to feed all the pumps is around 500 million BRLyear. The capital to implement coverage of the entire length of the channels with PV systems is estimated at an additional 2.0 billion BRL (11% of the transposition project’s CAPEX or 4 years of electricity cost). To convert amounts to US Dollars, a general rate of (5.00 *BRL*/1.00 *USD*) may apply.

## Conclusion

The lack of water has been a major obstacle to the human development of millions of Brazilians in the 21st century^6^. The Integration Project offers an efficient and structured solution to increase the water supply, providing relief to a population and an entire region suffering from drought. The primary objective of this transposition project is to supply the population with water. By reducing evaporation losses, the project will ensure greater water availability at the destination with the same amount of energy used in pumping, effectively improving the system’s energy efficiency^66–70^.

The use of free water surfaces to install floating PV arrays is a modern and sustainable C-free solution in the water-energy nexus. Electric vehicles of the maintenance fleet will help further reduce the project’s carbon footprint, making it more sustainable and environmentally friendly. Water savings of this solution will enhance the PISF’s main goals of supplying the deprived Northeast population, improving their quality of life.

To meet the power demand of the current stage pumping stations, PV systems must cover less than 20% of the channels. However, to reduce the evaporation loss, it is desirable to cover the entire length of the channels saving a daily additional 25,000 $m^3_{Water}$ to supply the population, and, generating 1,200 GWh yearly

The energy surplus generated from the entire coverage of the channels can be stored in battery stations (BESS) to compensate for the solar inconsistency and variability and ensure energy quality. This will contribute to supply the demand of low/no radiation time. Otherwise, this energy surplus will feed the electric grid to supply the National Integrated System (SIN), which contributes to mitigating the newest peak of demand that occurs in the summer between 10 a.m. and 4 p.m. according to data from the National Electric System Operator (ONS)^71^. This will avoid the use of thermal power plants that produce greenhouse gases and increase electricity costs, in addition to being in line with the Ministry of Mines and Energy’s ten-year planning^72^ which intends to reach the installed solar power plants capacity of 14.5 $\overline{GW}$ in 2030 (4.5% of the country’s electric matrix), and with Brazil’s GHG emission reduction commitments^73^ at the United Nations Conferences on Climate Change (UNFCCC), COP21 (Paris) and after^74^.

The concept of “solar canals” has been gaining traction around the world as climate change increases the risk of drought in many water-scarce regions^75^. The technology for PV installations is current and available. The scale is technically feasible, as India has shown^76^.

### Final thoughts

Evaluating the local solar radiation and environmental impacts, the same technology can be applied worldwide to irrigation channels in places where water is lost through evaporation. Asia already applies coverage of water channels, lakes, and, ponds with solar panels. Other continents, especially Africa, South America, and the Pacific Islands can also take advantage of this technology in the water-energy-food nexus, benefiting the local population. Despite the intensive capital costs required, the saving of water and power makes these projects feasible. The waterways would, in a sense, make PV panels water-cooled, boosting their efficiency. Energy and water co-benefits from covering channels with PV panels. Solar-paneling channels would not only produce renewable energy, it would run the water system itself^46,47^. These projects are fully aligned to the United Nations Development Programme (UNDP) –2030 Agenda for Sustainable Development–, Sustainable Development Goals (SDG) Nr. 6, 7, 8 and 9^77,78^. The use of PV-FPP will reduce emissions of ${CO_{2}}$, and other GHG, by electricity generation from fossil fuels, helping to combat climate changes and the impacts of the global warming process; SDG Nr. 13 (Climate Action). As the atmospheric pollution is only getting worse, it requires application development or PV expansion to avoid unnecessary environmental impacts. This does not need direct funding, but a suitable policy^79^ that serves humanity^80^.

## Data Availability

All data generated or analysed during this study are included in this published article.
